# Gastritis

**Published:** 1872-09-15

**Authors:** 


### Clinical Reports.

[From the service of Prof. N. S. Davis in medical wards of Mercy Hospital.]

### GASTRITIS.

FROM NOTES BY WM. H. B.

*Gentlemen :* This man I have not seen before, and you have heard his statement to the effect that he came to this country a little more than a year ago, when he went to work in the hay and harvest field, laboring harder than he was used to. He states that during the harvest he drank excessively of milk and water. About last Christmas he felt a burning, disagreeable pain in his stomach, and had attacks of vomiting soon after eating. Though he dates this uncomfortable feeling about Christmas, I presume, had he have paid particular attention, he would have noticed it in September.

In the morning, when his stomach is empty, he feels very well, and feels as though he could eat a good large breakfast ; but almost as soon as he gets his food down, he recognizes the old pain, and after two or three hours it is generally thrown off. Now mark this particular symptom. The food is soon thrown off, and the pain is of a burning, heating character. When I asked him what he vomited, he said if he vomited soon after his food was taken into the stomach, he vomited but his food, which is very sour ; but when it is retained for some time, there is much more thick mucus. Gentlemen, we could easily diagnose this case as that of indigestion ; but this would not benefit us much unless we know the exact state of the stomach which causes the failure in digestion.

The simple failure of the stomach to digest the food may arise from either deficient secretion of gastric juice, some grade of inflammation affecting the mucous membrane, or from cancerous disease.

The first class of cases consists of a want of the peristaltic motion, and insufficient secretion of the gastric juice.

Now if this were the case with this patient, after taking food he would feel a heavy weight in his stomach, with frequent belching of wind or gases.

Patients of this class seldom have vomiting, the bowels remaining torpid, the food lies like a load for two or three hours, undergoing more or less fermentation, when it is worked off, and the patient again feels quite comfortable. This is the most frequent class you will be likely to meet with. A history of this man shows you that it does not belong to this category, as there is a soreness on pressure over the stomach, and a burning pain, with vomiting.

In the second class, where there is an inflammation of the follicles, there is a sense of uneasiness felt before the meal is fairly finished, frequently obliging the patient to get up from the table and go out to vomit.

When the inflammation affects the mucous membrane, generally in a low chronic form, it does not allow the gastric juice to be secreted, but it causes the formation of an excess of mucus, generally of an extremely acid character. Consequently food not only produces immediate distress, which grows worse and worse until vomiting is induced, but the matters vomited are sour, and often acrid.

There is one class belonging to this category which will give you a different train of symptoms, and that is when the follicles are exclusively involved. In such cases the patient is apt to vomit within thirty or forty minutes after having taken food ; but you will not find an atom of food in the matters vomited, that which is thrown up being, in some cases, a thin watery mucus, and, in others, as much as a teacupful of thick, ropy mucus. Now, in such cases, both mucus and gastric juice are secreted in such large quantities that it hurries the solution and discharge of the food from the stomach into the intestines. In these cases the patient does not feel much uneasiness until time enough has elapsed for the food taken to have become dissolved and passed out of the stomach; but the gastric secretions continuing their accumulation, soon occasion sufficient distress to induce vomiting, when these secretions alone are ejected, sometimes sharply acid, at others tasteless.

Some of this class of patients vomit a quantity of the gastric secretion every morning before taking food. Such cases are often called pyrosis, or waterbrash. The distress accompanying such cases is more a gnawing or craving than pain.

From these general remarks you will recognize the case before you as that of a diffusive inflammation, affecting both the follicles and mucous membrane of the stomach.

Before we decide positively, however, on the diagnosis of this case, we should allude briefly to the diagnostic symptoms of cancer of the stomach. The fact is, cancerous disease rarely causes such smarting and burning pain as chronic inflammation. The favorite seat of cancer is at the pyloric orifice of the stomach, and the food is retained for an hour or more before the most severe pain is experienced. There is another difference—the cancerous patient seldom vomits more than a small portion of his food, it being usually digested and absorbed in part, leaving but little to be ejected with the ropy mucus that is generally vomited in from one to two hours after taking food. When cancerous disease of the stomach has existed for several months, the patient will often go from six days to two weeks without a passage from the bowels, and still the abdomen will be lank and empty.

Now, gentlemen, when you find a patient past middle life, free from fever, vomiting mostly mucus an hour or more after eating; bowels never moving except by medicine, yet the abdomen lank and empty; with gradual emaciation; you may be quite sure you have cancer of the pylorus.

In chronic inflammation the bowels are generally distended with gases. There is also another item which will help you out in your diagnosis. In cancer the disease comes on slower, and the patient cannot tell you positively when he first began to feel unwell; whereas in chronic inflammation the patient will tell you pretty promptly when his gastric troubles commenced.

If it be true that the patient before you has a low grade of chronic inflammation of the mucous membrane of the stomach, the longer he continues to dose himself with active physic, under the popular notion that he is bilious, the worse he will get. The principle of treatment should be to give only the most bland and simple articles of nourishment, and such medicines as are calculated to allay the morbid sensitiveness of the inflamed surface. One of the best items of nourishment is a mixture of three parts of sweet milk and one of lime-water. At first it should be given in doses of one or two tablespoonfuls, and repeated every hour. The lime-water neutralizes the excess of acid in the secretions of the stomach, and aids in preventing the coagulation of the casein of the milk until it is absorbed. Once or twice a day a small quantity of thin porridge, made of milk and wheat flour, may be given in addition to the other. Also, occasionally a tablespoonful of animal broth may be allowed. For medicine we will give him, for the first three or four days, the following prescription:

| | | |
|---|---|---|
| ℞ | Sub. nit. bismuth, | 40 grs. |
| | Pulv. ipecac, | 4 grs. |
| | Hydrg. chlorid. mite, | 4 grs. |
| | Pulv. Doveri, | 20 grs. |

Mix, divide into eight powders, and take one before each meal-time, and at bed-time.

After three or four days the powder may be superseded by a pill containing one grain of extract of hyoscyamus and one-third of a grain of nitrate of silver. In some cases a pill composed of one grain each of extract of hyoscyamus and sulphate of iron, taken just before each meal, produces a very beneficial effect. But no kind of medication will succeed in these cases without the most rigid care in relation to the food and drinks.

